# Transfer entropy for synchronized behavior estimation of interpersonal relationships in human communication: identifying leaders or followers

**DOI:** 10.1038/s41598-019-47525-6

**Published:** 2019-07-29

**Authors:** Kenji Takamizawa, Masahiro Kawasaki

**Affiliations:** Department of Intelligent Interaction Technology, Graduate School of Systems and Information Engineering, University of Tsukuba, 1-1-1, Tennodai, Tsukuba-shi, Ibaraki 305-8573 Japan

**Keywords:** Human behaviour, Applied mathematics

## Abstract

A person’s behavioral rhythms are synchronized spontaneously and unconsciously with those of other people, which often have positive effects, such as facilitating cooperation on tasks and promoting empathy for others. Although synchronization is induced by mutual interaction, it is unclear whether both individuals have the same influence. Is there a division of roles, in which some people are leaders and some followers? To address this, we calculated the transfer entropy (TE) of behavioral rhythms in a two-person cooperative tapping task, which provides an estimate of the direction of information propagation between two systems. We used TE to identify the causal relationship between two people (leader and follower); that is, the significant differences in the TE from one partner to another and vice versa. In this study, if there was a high TE from one individual (e.g., participant A) to the other individual (e.g., participant B), we defined participant A as the leader group and B as the follower group. First, using computer simulations, the programs which tapping intervals were almost independent with or were almost same with those of the partner programs were identified as the leader or follower, respectively, thereby confirming our hypothesis. Second, based on the results of the human experiment, we identified the leader and follower in some groups. Interestingly, the leader group showed a high systemizing quotient, which is related to communication deficits in developmental disorders such as autism. The results are consistent with participants’ subjective impressions of their partners. Our methods can be used to estimate the interpersonal division of roles in complex human communications.

## Introduction

In a one-on-one communication link, people more or less strive to compromise and match the thoughts and behaviors of the other party. For example, in your office, you are concerned about your boss, while he might be also concerned about you. Such communication involves mutual interactions or synchronizations. Synchronization involves several cognitive functions; prediction of the behavior, intentions, and feelings of others^1,2^; and adaptation to errors between the prediction and reality^3^. We experience such synchronizations unconsciously and spontaneously in several social situations, such as hand-clapping in a musical concert^4^, speech rhythm^5,6^ and nodding^7^ in daily conversation. Importantly, it induces positive effects on interpersonal communication, such as affiliation^8^, empathy^9^, and good impression^10^. It is also important in building relationships within the group, which can lead to a higher performance on cooperative tasks^11^ and compassion for the others^12^. However, the two parties contributing to the synchronization do not always have the same workload. Sometimes, there is a bias, whereby one is free to act, and the other has to keep pace.

Previous studies of the behavioral characteristics of human communication have examined simple synchronization of behavior between individuals, such as finger tapping^13^ and walking together^14^. This type of behavioral synchronization requires a temporal correlation between individual rhythms; that is, the rhythms must occur simultaneously^15^. Individual rhythms are dependent on the individual cognitive processing (e.g., sensory-motor brain processing) and are independent of each other; thus, synchronization occurs randomly and unconsciously^16,17^. Furthermore, there are large individual differences in the abilities and strategies for predicting others’ rhythms and adapting to them. Therefore, behavioral synchronization might reflect in individuals’ different characteristics (i.e., strategies). However, the relationship between synchronization and their individual characteristics has yet to be clarified.

This study aimed to clarify the leader and follower relationship between two individuals. Some previous studies of leader-follower interactions involved predetermining the roles of the leader and the follower and investigating their behaviors in the experimental conditions^18,19^. Other studies have clearly shown the mechanisms of the leader-follower relationship by using a lagged cross-correlation analysis, even when the roles were not predetermined^20,21^. However, only the lagged cross-correlation analysis is difficult to identify the detailed causality between the leaders and followers, due to some limitations such as assumption of the stationarity and the normality. Therefore, the mechanism of the division of roles, which occurs unconsciously in a natural state without the consideration of the above assumptions, remains unclear. To address this, the present study attempted to apply transfer entropy (TE)^22,23^ between two participants for their behavioral data on cooperative tasks that required them to synchronize their behavior. TE is an index, which reflects the causal information flow between two systems, such as the relationship between the heart rate and breathing rate^22^ and the relationship between the activities of distant brain areas^24^. Generally, when there is a flow of information, the TE from the source system to the receiving system increases. In this study, if there was a high TE from one individual (e.g., participant A) to the other individual (e.g., participant B), we defined participant A as the leader and participant B as the follower. We predicted that the follower would try to adjust to the partner’s rhythm, whereas the leader would not be affected by the follower’s rhythm.

**Figure 1:**
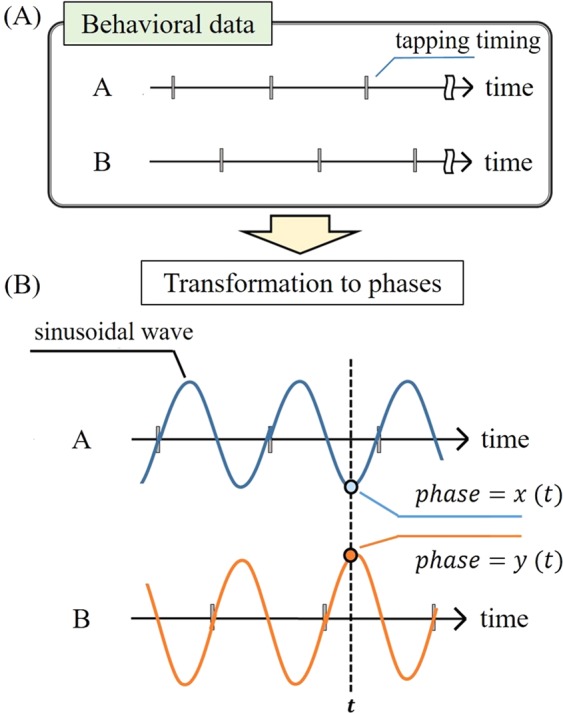
Illustration of the transformation from behavioral data (**A**) to continuous phase data (**B**) to calculate the TEs.

We used a cooperative alternate-tapping task^17,25^, in which pairs of participants were required to take turns pressing a keyboard key, with each participant matching the time interval set by the partner. This study consisted of two parts: computer simulations (participants were PC programs, that is, virtual humans) and human experiments (participants were real humans). To apply the tapping interval data to the TE analyses, we transformed the discrete tapping timing data to successive phase data (from -pi to pi; Fig. 1); that is, each participant’s tapping intervals were transformed into a one-cycle sinusoidal wave. We used the time-course phase data to calculate the TE.

## Method

### Computer simulation

We created the computer programs with two parameters for the tapping time intervals. One was leader-like randomness, in which the interval was dependent on the normal distribution (mean = 1.0; SD = 0.1), independent of the previous interval of the partner program. The second was a follower-like adaptation, in which the interval was the same as the previous interval of the partner program. For instance, in this study, a PC program with time intervals consisting of 80% randomness and 20% adaptation was described as PC80 (i.e., the number indicates the percentage of randomness). We created four types of PC programs: PC80, PC60, PC40, and PC20. We hypothesized that PC programs with higher randomness (e.g. PC80) would be identified as leader-type programs, whereas those with higher adaptation (e.g. PC20) would be identified as follower-type programs.

### Estimation of transfer entropy

To apply the tapping interval data in this study, we transformed the discrete tapping interval data into successive data. For each participant, the interval between one tap and the next one was defined as one period and was transformed into a single-cycle sinusoidal wave. The phases (from -pi to pi) for time ***t*** were defined as ***x***(***t***)and ***y***(***t***) for two participants (***X***, ***Y***), respectively. The first 10 taps were excluded from the analyses because the timing for some of the initial trials was unstable.

***TE***(***X***, ***Y***, ***τ***), representing the entropy from ***X*** to ***Y*** for the time lag ***τ***, was calculated with the following formula^22^:

1 $$TE(X,Y,\tau )=\frac{1}{T}{\int }_{0}^{T}\,{\mathrm{log}}_{2}\frac{{P}_{Y|YX}(y(t+\tau )|y(t),x(t))}{{P}_{Y|Y}(y(t+\tau )|y(t))}dt$$

TE was calculated by averaging the data for the time window, which was defined as ***T*** = 270 sec, controlling for task conditions. To estimate the information flow, we compared ***TE***(***X***, ***Y***, ***τ***) and ***TE***(***Y***, ***X***, ***τ***) using permutation tests (1,000 times). Causal information flow was indicated by significant differences between the TEs in the permutation tests (p < 0.05). For example, there was an information flow from ***X*** to ***Y***, provided that formula (2) was accepted.

2 $$TE(X,Y,\tau ) > TE(Y,X,\tau )$$

The denominator, ***P***_***Y***|***YX***_(***y***(***t*** + ***τ***)|***y***(***t***), ***x***(***t***)) in formula (1) was transformed as follows.

3 $${P}_{Y|YX}(y(t+\tau )|y(t),x(t))=\frac{{P}_{YYX}(y(t+\tau ),y(t),x(t))}{{P}_{YX}(y(t),x(t))}$$

The denominator, ***P***_***YYX***_(***y***(***t*** + ***τ***), ***y***(***t***), ***x***(***t***)), in formula (3) was calculated using the Kernel density estimation as follows.

4 $${P}_{YYX}(y(t+\tau ),y(t),x(t))=\frac{1}{N-\tau }\sum _{t^{\prime} =1}^{N-\tau }\,\Theta (r-{\Vert \begin{array}{c}y(t^{\prime} +\tau )-y(t+\tau )\\ y(t^{\prime} )-y(t)\\ x(t^{\prime} )-x(t)\end{array}\Vert }_{\max })$$

5 $$\Theta (z)=\{\begin{array}{ll}1, & if\,z\ge 0\\ 0, & ifz < 0\end{array}$$

***N*** indicates the number of data points, ***r*** indicates the inclusion radius for the neighborhood search, and ||…||_*max*_ represents the maximum distances.

Formula (4) depends on the time lag ***τ*** and the inclusion radius ***r***. In this study, ***τ*** was fixed at the average time interval between the two participants’ taps and ***r*** was defined using the computer simulations. In the simulations, when we fixed ***r*** from 2 to 50, the best value for dissociating the TEs was about 10.

### Permutation test

To evaluate the significances of the differences between TEs, we used the permutation tests. First, we calculated the TEs by using the time intervals between one tap and the next one for all tapping data. The difference between the TEs was defined as the raw result. Second, we randomly rearranged the time intervals and calculated the TEs using the rearranged time intervals. The difference between the TEs was defined as the rearranged result. The rearranged analyses were repeated 100 times. Finally, we counted the number of times the raw results was over the rearranged ones per 100 times.

### Alternate tapping task

Twenty-four healthy right-handed male volunteers (21.5 ± 3.0 years) participated in the experiments. The study was approved by the Ethical Committee of the University of Tsukuba and was in accordance with the Declaration of Helsinki. Moreover, all methods were performed in accordance with the relevant guidelines and regulations. All participants gave written informed consent before participating in the study.

The participants were divided into eight groups of three participants. Within each group, each participant completed four experimental sessions with the other two participants. Therefore, the total number of pairs was 24.

Throughout the task, the participants wore earphones and sat in a chair opposite their partners. In front of each participant, there was a computer monitor and keyboard. The participants were required to keep their bodies still and fix their eyes on the white fixation points on the computer display. The inter-session intervals were for at least 1 minute.

In two sessions, the pairs of participants tapped their respective keys alternately with their right index fingers^25^. When a participant tapped, a sound (either “do” or “shi” on a piano scale) was presented for 100 msec through both right and left headphones. When the partner tapped, the other sound (either “shi” or “do”) was presented through both right and left headphones. The same procedure was followed in the other two sessions. In both sessions, the participants were instructed to tap the key at a time interval equal to that of their partners. The tapping rhythms were not predetermined or directed. The tasks continued for 300 seconds.

### AQ, EQ, and SQ

The AQ is a self-report questionnaire consisting of 50 statements about the symptoms of autism spectrum disorder^26^. The scale consists of five autistic domains: social skills, communication skills, imagination, attention to detail, and attention switching. The EQ is a self-report questionnaire consisting of 80 statements about empathy, including both cognition and affect^27^. It defines empathy as the ability to experience an appropriate emotion in response to another’s behavior and the ability to understand others’ emotions. The SQ is a self-report questionnaire consisting of 80 statements about systemizing, which is defined as the ability to understand and analyze patterns and rules in systems^28^.

The items on the AQ, EQ, and SQ have four response options: “definitely agree,” “slightly agree,” “slightly disagree,” and “definitely disagree.” All questions are mandatory. Each question scores one point and the maximum total scores are 50 for the AQ and 80 for the EQ and SQ.

### Post-experimental questionnaire

After the tasks, the participants were asked to answer ten questions about the tasks (Table 1). The participants chose from five response options: “strongly agree,” “slightly agree,” “neither agree nor disagree,” “slightly disagree,” and “strongly disagree.”

**Table 1:** Post-experimental questionnaires about the tasks.

Q1	“To what extent did you feel angry when your rhythms were not synchronized?”
Q2	“To what extent did you regret it when your rhythms were not synchronized?”
Q3	“To what extent did you proactively try to keep pace with your partner when your rhythms were not synchronized?”
Q4	“To what extent were you concerned about your partner’s behaviors during the experiments?”
Q5	“To what extent did you focus on maintaining your own rhythms rather than keeping pace with your partner’s?”
Q6	“To what extent did you feel that the desynchronization of your rhythms was due to your partner?”
Q7	“To what extent did you feel that it gradually became easier to synchronize with your partner’s rhythm?”
Q8	“To what extent did you feel happy when your rhythms were synchronized?”
Q9	“To what extent could you understand the rhythm of your partner’s tapping?”
Q10	“To what extent did you feel that your personalities were evident in your tapping?”

## Results

### Computer simulations

First, we performed a computer simulation using two different PC programs to virtually complete the alternate tapping task, in order to confirm the usefulness of TE in estimating the causal information flow. TE depends on the time lag ***τ*** and the inclusion radius ***r***. In this study, the ***τ*** was fixed to the average time interval between the two participants’ taps, and ***r*** varied from 2 to 50. To identify the optimal ***r***, there were 6 simulations with tasks between the different programs (PC80-PC20, PC80-PC40, PC60-PC20, PC80-PC60, PC60-PC40, and PC40-PC20). Each simulation was conducted 100 times.

Table 2 shows the ratio of pairs in which TE from a large-randomness PC (e.g., PC80) to a small-randomness PC (e.g., PC20) was larger than TE from a small-randomness PC to a large-randomness PC in each simulation; that is, the ratio of pairs in which our hypothesis was confirmed. The simulations with the large-different randomness PCs (i.e., PC80-PC20, PC80-PC40, and PC60-PC20) showed 95% more than ratios described above, regardless of ***r***. Particularly, the large-randomness PC was identified as the source of information transfer. Moreover, the simulations with small-different randomness PCs (i.e., PC40-PC20, PC60-PC40, and PC80-PC60) showed 70% more than the ratios described above, although the ratios were smaller than those of the simulation with the large-different randomness PCs. In such a case, the ratios were high when the ***r*** was under 10.

**Table 2:** Ratios of pairs in which TE from the large-randomness PC to the small-randomness PC was larger than TE from the small-randomness PC to the large-randomness PC in each computer simulation manipulating *r*.

*r*	2	4	6	8	10	12	14	16	18	20	…	25	…	30	…	40	…	50
PC80 vs. PC20	1.00	1.00	1.00	1.00	1.00	0.99	0.99	0.99	0.99	0.99		0.98		0.99		0.98		0.98
PC80 vs. PC40	1.00	0.99	0.98	0.96	0.96	0.95	0.94	0.94	0.94	0.94		0.94		0.94		0.94		0.95
PC80 vs. PC60	0.86	0.84	0.83	0.79	0.74	0.72	0.68	0.66	0.66	0.66		0.69		0.69		0.71		0.71
PC60 vs. PC20	0.99	0.99	1.00	1.00	1.00	0.99	0.99	0.99	0.98	0.98		0.98		0.98		0.99		0.99
PC60 vs. PC40	0.78	0.86	0.87	0.85	0.85	0.87	0.86	0.85	0.83	0.81		0.79		0.80		0.80		0.81
PC40 vs. PC20	0.78	0.82	0.84	0.86	0.84	0.82	0.80	0.80	0.80	0.78		0.80		0.79		0.79		0.81

Next, we assessed the differences between TE from the large-randomness PC to the small-randomness PC and the TE vice versa, by running the permutation test 100 times. Table 3 shows the ratio of pairs in which TE from a large-randomness PC to a small-randomness PC was significantly larger than TE vice versa (p < 0.05) in each simulation. In all simulations, the ratios were the maximum when the ***r*** was close to 20. However, misidentifications (the numbers in parentheses in Table 3 indicate an opposite association) were found when the ***r*** was over 12. The maximum and minimum ratios were found in the simulation with large-different randomness PCs (PC80-PC20) and small-different randomness PCs (PC40-PC20), respectively, independent of ***r***.

**Table 3:** Ratios of pairs in which TE from the large-randomness PC to the small-randomness PC was significantly larger than TE vice versa (p < 0.05) in each computer simulation manipulating *r* (the parentheses indicate the opposite of large and small).

*r*	2	4	6	8	10	12	14	16	18	20	…	25	…	30	…	40	…	50
PC80 vs. PC20	0.14	0.26	0.41	0.61	0.80	0.86	0.88	0.89	0.89	0.90		0.90		0.91		0.88		0.78
PC80 vs. PC40	0.00	0.02	0.11	0.23	0.38	0.55	0.66	0.72 (0.01)	0.74 (0.01)	0.76 (0.02)		0.74 (0.01)		0.66 (0.01)		0.62		0.52
PC80 vs. PC60	0.00	0.00	0.02	0.06	0.11	0.18	0.26	0.30	0.38 (0.02)	0.43 (0.02)		0.41 (0.02)		0.33 (0.02)		0.30		0.20
PC60 vs. PC20	0.01	0.03	0.05	0.19	0.35	0.55	0.67	0.71	0.75	0.77		0.74		0.74		0.60		0.50
PC60 vs. PC40	0.00	0.00	0.01	0.07	0.19	0.26 (0.01)	0.32 (0.02)	0.37 (0.02)	0.40 (0.02)	0.42 (0.02)		0.41 (0.02)		0.38 (0.02)		0.32 (0.02)		0.28 (0.02)
PC40 vs. PC20	0.00	0.00	0.02	0.06	0.11	0.18	0.26	0.30	0.38 (0.02)	0.43 (0.02)		0.41 (0.02)		0.33 (0.02)		0.30		0.20

Moreover, we performed computer simulations using pairs of the same type of PC programs (e.g. PC80 versus PC80). Table 4 shows the ratio of pairs in which TEs were significantly different (p < 0.05) in each simulation. The simulations were assumed to show no significant differences, since the two PC programs were almost the same. The assumption was satisfied when the ***r*** was under 10. The maximum and minimum ratios were found in the simulation with large randomness PCs (PC80-PC80) and small randomness PCs (PC20-PC20), respectively.

**Table 4:** Ratio of pairs in which TEs were significantly different (p < 0.05) in each computer simulation manipulating *r* using the pairs of the same type of PC programs.

*r*	2	4	6	8	10	12	14	16	18	20	…	25	…	30	…	40	…	50
PC80 vs. PC80	0.00	0.00	0.00	0.01	0.08	0.21	0.30	0.33	0.38	0.44		0.43		0.37		0.32		0.26
PC60 vs. PC60	0.00	0.00	0.00	0.02	0.06	0.12	0.18	0.22	0.30	0.33		0.32		0.27		0.19		0.11
PC40 vs. PC40	0.00	0.00	0.01	0.01	0.03	0.09	0.14	0.18	0.19	0.22		0.23		0.23		0.16		0.12
PC20 vs. PC20	0.00	0.00	0.00	0.00	0.00	0.02	0.03	0.06	0.05	0.07		0.07		0.05		0.03		0.02

Next, we performed the simulation by manipulating the time lag ***τ***, from 0.5 to 4.0 sec in increments of 0.5, instead of the average tapping intervals. The ***r*** was fixed to 10, based on the results of the above simulation. Each simulation was performed 100 times. The ratios, which confirmed the hypothesis, are shown in Table 5. TE could evaluate the relationship when ***τ*** was approximately between 1.0 and 2.0. In contrast, when ***τ*** was both high (e.g. over 3.0) and low (e.g. under 0.5), the ability of the evaluation decreased.

**Table 5:** Ratios of pairs in which TE from the large-randomness PC to the small-randomness PC was larger than TE vice versa in each computer simulation manipulating *τ*.

τ	0.5	1.0	1.5	2.0	2.5	3.0	3.5	4.0
PC80 vs. PC20	0.98	1.00	1.00	1.00	1.00	0.99	1.00	0.99
PC80 vs. PC40	0.94	0.96	0.95	0.97	0.96	0.96	0.94	0.92
PC80 vs. PC60	0.70	0.71	0.74	0.76	0.74	0.74	0.73	0.70
PC60 vs. PC20	0.95	0.98	0.99	0.99	0.99	0.96	0.97	0.96
PC60 vs. PC40	0.75	0.78	0.81	0.79	0.78	0.76	0.76	0.71
PC40 vs. PC20	0.69	0.83	0.86	0.86	0.83	0.83	0.81	0.72

### Human experiments

Second, we conducted the human experiments between pairs of individuals (total number of pairs = 96; total number of participants = 24) to determine whether they adopted leader or follower roles, leading to synchronized behavior during the alternate cooperative tapping task. According to the results of the computer simulations, the ***τ*** was fixed to the average time interval between the two participants’ taps and ***r*** was fixed to 10 in the TE analyses for the human experiments. The participants-averaged time interval in this study was 1.15 ± 0.34 sec. The maximum and minimum time intervals were 2.63 sec and 0.52 sec, respectively.

To examine the relationship between leaders and followers and their personality traits, participants completed three questionnaires about human communication: the autism-spectrum quotient (AQ)^26^, the empathy quotient (EQ)^27^, and the systemizing quotient (SQ)^28^. The AQ is an index that measures the symptoms of autism spectrum disorder, which is characterized by deficits in communication skills and the presentation of restricted behaviors^26^. The EQ and SQ measure empathetic and systemizing skills, respectively. Individuals who show high EQ scores are highly capable of predicting others’ emotions and intentions. In contrast, individuals with high SQ scores are good at understanding the natural laws and patterns of a phenomenon. The results showed that the average AQ, EQ, and SQ scores in this study were 21.5 ± 6.29, 30.6 ± 9.92, and 27.8 ± 11.83, respectively.

**Figure 2:**
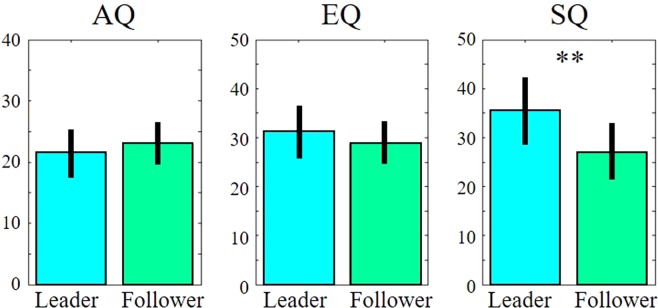
Average AQ, EQ, and SQ scores of the leader and follower groups. The error bars indicate the standard error means. The double asterisks denote significant differences (p < 0.01).

The TE analyses identified the causal relationships between pairs of participants to identify leaders and followers; that is, the analyses examined significant differences (p < 0.05) between the TE of one participant and that of another participant, and vice versa, in 61 of the 96 pairs. The SQ scores of the leader group were significantly higher than those of the other groups (t = 3.71; p < 0.01), whereas the AQ scores (t = −1.16; p = 0.25) and EQ scores (t = 1.34; p = 0.18) showed no significant differences (Fig. 2).

The identification of participants as leaders and followers did not seem to be affected by other factors such as the order of play and the type of cue sound (same or different sound). Of the 61 pairs that showed significant differences between TEs, 31 pairs played first and 30 pairs played second, and 28 pairs used the same sounds and 33 pairs used different sounds. These results suggest that the identification of the leader or follower was not influenced by who played first or by the ease of separating oneself from others.

**Figure 3:**
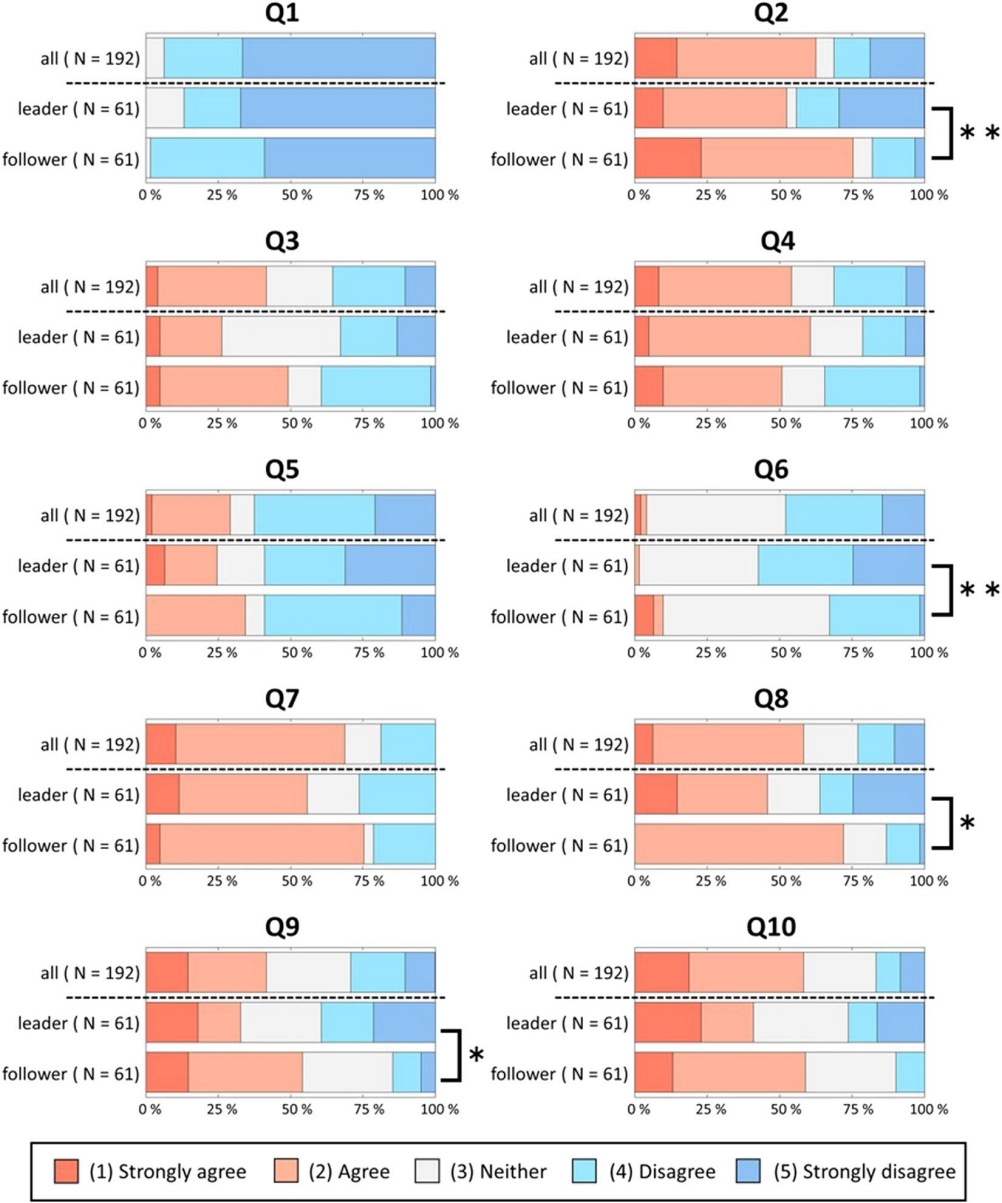
The percentages of “applicable,” “neither applicable nor not applicable,” and “not applicable” responses to the post-experimental questionnaire about the participant’s feelings toward the task and the partner.

The post-experimental questions about the division of roles in the task also showed significant differences between the leader and follower groups (Fig. 3). Approximately 75% of the follower group responded “agree” to the question “Q2: To what extent did you regret it when your rhythms were not synchronized?” and “Q8: To what extent did you feel happy when your rhythms were synchronized?” On the other hand, approximately 40% of the leader group responded “disagree” to these questions (Q2, z = 3.42, p < 0.01; Q8, z = 2.18, p < 0.05; Wilcoxon sign-rank test). Furthermore, there were significant differences between the leader and follower groups in the responses to the following two questions: “Q6: To what extent did you feel that the desynchronization of your rhythms was due to your partner?” and “Q9: To what extent could you understand the rhythm of your partner’s tapping?” (Q6, z = 3.61, p < 0.01; Q9, z = 2.52, p < 0.05). The responses to the other questions are shown in Fig. 3. The follower group made an effort to work out the partner’s pattern in the task and had a sense of cooperation. In contrast, the leader group did not show such tendencies; particularly, they were not aware of the partner during the task.

## Discussion

The TE analyses in this study could identify leaders and followers in the synchronization of behavior on cooperative tasks. First, we performed computer simulations with programs that differed in their patterns of synchronization: the leader was always free to act (i.e., large randomness) and the follower was always keeping pace (i.e., small randomness). We hypothesized that TE from the leader-like behavior to the follower-like behavior would be large. The hypothesis was confirmed by the computer simulations, which showed clear dissociations between the leader and follower types (Tables 2 and 3). In particular, when the interpersonal relationship of the pairs was evident, that is, the randomness of the two PC programs was considerably different, the TE was significantly different.

In contrast, in pairs that showed no difference in TEs, both individuals acted as leaders or followers. In the computer simulation using the similar-type programs (i.e., the randomness of the two programs were similar or the same), the TEs were not significantly different. In the case of both leaders, the two individuals might have behaved selfishly and not affected by each other. In the case of both followers, both individuals might have had an effect on each other’s rhythms, which may have induced behavioral synchronization. Either case induces small asymmetries in the behaviors of both individuals, which indicated no differences between TEs from either direction.

Moreover, when the behaviors of two individuals were similar, there were few cases in which the relationship between large and small TEs was reversed, contrary to the hypothesis, as shown in Table 3. However, the erroneous discrepancies were excluded by the permutation tests.

The TEs may have been affected by the obscure variable ***r*** (the inclusion radius for the neighborhood search; see Method section)^22,23^. A previous study proposed that a large amount of reference data would be needed to calculate TE^22^. Indeed, TE showed large fluctuations right after the start of a trial because of the small number of data points during the initial trials. We calculated the TEs with the variable *r* fixed from 2 to 50. When *r* was fixed at 20, the TEs were clearly dissociated and showed significant differences between individuals. Although the resolutions of the dissociations were high, there were some errors, in which the values were reversed for the leaders and followers. In contrast, when *r* was fixed at 10, the TEs were clearly dissociated, although not as much as when it was fixed at 20, and showed no errors of the dissociations. Therefore, we fixed *r* at 10 in the TE analyses for the human experimental data.

Further, we also calculated the TE by manipulating the time lag *τ*. The results showed more identifications of the leader and follower when *τ* was between 1.0 and 2.0. The time ranges were almost the same as the average tapping intervals between the individuals in human pairs (1.15 ± 0.34 sec). In contrast, when *τ* was high, the TE analyses showed the effect of the current tapping on the two previous tappings. Moreover, when *τ* was low, the TE analyses showed no significant effect of the current tapping on the next tapping. Therefore, we used the average time interval between two participants’ taps as the *τ* in the TE analyses for the human experimental data.

In the computer simulations, we defined the lead-like and follower-like programs based on the ratios between the leader-like randomness and follower-like adaptation. Our results showed the clear dissociation between the leader and follower by using TE for the above programs. In relation to these programs, previous studies have developed the Adaptation and Anticipation Model (ADAM), which was based on the reactive error correction processes and predictive temporal extrapolation processes of sensory-motor synchronization^3^. Although the study has not dealt with the leader-follower relationships, the ADAM could simulate one’s own and the partner’s actions. Therefore, future study might be able to simulate the leader-like and the follower-like behaviors by modulating the adaptation and anticipation parameters.

Second, the human behavioral experiments confirmed the validity of the TE analyses of the aforementioned variables of the alternate tapping task in the identification of interpersonal relationships. We identified the causal relationships between pairs of participants to identify leaders and followers in 61 of the 96 pairs. In the additional analyses with 20 as the variable *r*, we identified leaders and followers in 67 of the 96 pairs. Although the number was higher than that in case of 10 as the variable *r*, there were possible errors according to the computer simulations. Moreover, we confirmed no differences in the AQ, SQ, and EQ scores between these analyses (*r* = 10 and *r* = 20). When *r* was 20, the SQ scores of the leader group were significantly higher than those of the other groups (t = 4.14; p < 0.01), whereas the AQ scores (t = −1.38; p = 0.17) and EQ scores (t = 0.91; p = 0.36) showed no significant differences.

The successful identification of the leader and follower might be due to the different SQ scores in pairs being large or small. There were significant differences in the SQ scores between the leaders and followers in pairs that showed successful identification (i.e., significant differences of TEs). In contrast, there were no significant differences (t = 1.37; p = 0.17) in the SQ scores in pairs that showed no significant differences in TEs. These findings are consistent with those of the computer simulations, which indicated that TEs were identified when the type (e.g. randomness) of the two PC programs were largely different.

The alternate tapping tasks required the participants to predict both the system’s patterns and their partners’ behavioral patterns. The leaders’ high SQ scores could be related to the restricted, repetitive patterns of behavior that are characteristic of autism^28^. They systematically and automatically dealt with the tasks, but did not keep pace with their partners and tapped at their own pace. A study showed that individuals with autism experienced difficulty in adapting to the irregular rhythms of the others’ behaviors, and the difficulties were closely correlated with the scores of repetitive and regular behaviors in the psychiatric assessments of autism^25^.

In contrast, there were no significant differences in the EQ and AQ scores between the leaders and followers because the alternate tapping tasks might have only required the ability to predict others’ behavior and patterns rather than the abilities of empathy and social communication. A future study should adopt a psychiatric approach to clarify whether the TE analysis identifies individuals with autism as leaders. Taken together, our TE analyses demonstrate that behavioral synchronization is closely related to individuals’ personalities.

According to the analysis of the post-experimental questionnaire, the identification of the leader and followers might be related to the division of roles as well as SQ abilities. In Q6, the leaders did not feel that the desynchronization of their rhythms was due to their partners. That is, they might have realized the difficulties in adapting to others’ rhythms. Moreover, in Q2 and Q9, the leaders did not feel sorry that their rhythms were not synchronized and they could not understand the rhythm of their partner’s tapping. In contrast, the followers showed the opposite results. These findings suggested that the leaders might not be interested in their partners’ behaviors unlike the followers, who try to keep pace.

To estimate the causality between the 2 signals, previous studies have used the lagged cross-correlation^20,21^, Granger causality^29^, and so on. However, theses analyses were not applicable with our data. For example, the cross-correlation analyses need the assumption of the stationarity and the normality. Moreover, the Granger causality requires pre-verified models^30^, and therefore it is not suitable in cases with model-free measurements like this study. Therefore, this study applied TE as a model-free causality analysis.

The findings of this study might make contribution to the anticipation of the role division in social environments, since the low-level synchronization behaviour in this study may well be linked to higher-level cognitive functioning in social environments. In addition, because the classifications were related to the symptoms of autism spectrum disorder, our method could be useful for identifying situations in which individuals with autism can best demonstrate their abilities.
